# Definitions and potential health benefits of the Mediterranean diet: views from experts around the world

**DOI:** 10.1186/1741-7015-12-112

**Published:** 2014-07-24

**Authors:** Antonia Trichopoulou, Miguel A Martínez-González, Tammy YN Tong, Nita G Forouhi, Shweta Khandelwal, Dorairaj Prabhakaran, Dariush Mozaffarian, Michel de Lorgeril

**Affiliations:** Department of Hygiene and Epidemiology, School of Medicine, University of Athens, Greece; Hellenic Health Foundation, Athens, Greece; Department of Preventive Medicine and Public Health, School of Medicine, University of Navarra, Pamplona, Spain; CIBER-OBN, Instituto de Salud Carlos III, Madrid, Spain; MRC Epidemiology Unit, University of Cambridge School of Clinical Medicine, Box 285, Institute of Metabolic Science, Cambridge Biomedical Campus, Cambridge, CB2 0QQ UK; Public Health Foundation of India (PHFI), New Delhi; Centre for Chronic Disease Control (CCDC), New Delhi, India; Friedman School of Nutrition Science and Policy, Tufts University, Boston, MA USA; Laboratoire TIMC-IMAG CNRS UMR 5525, PRETA Cœur & Nutrition, and Faculté de Médecine, Université Joseph Fourier, Grenoble, France

## Abstract

The Mediterranean diet has been linked to a number of health benefits, including reduced mortality risk and lower incidence of cardiovascular disease. Definitions of the Mediterranean diet vary across some settings, and scores are increasingly being employed to define Mediterranean diet adherence in epidemiological studies. Some components of the Mediterranean diet overlap with other healthy dietary patterns, whereas other aspects are unique to the Mediterranean diet. In this forum article, we asked clinicians and researchers with an interest in the effect of diet on health to describe what constitutes a Mediterranean diet in different geographical settings, and how we can study the health benefits of this dietary pattern.

**Electronic supplementary material:**

The online version of this article (doi:10.1186/1741-7015-12-112) contains supplementary material, which is available to authorized users.

## Mediterranean diet: what it is, what it does, how it works

**Figure 1:**
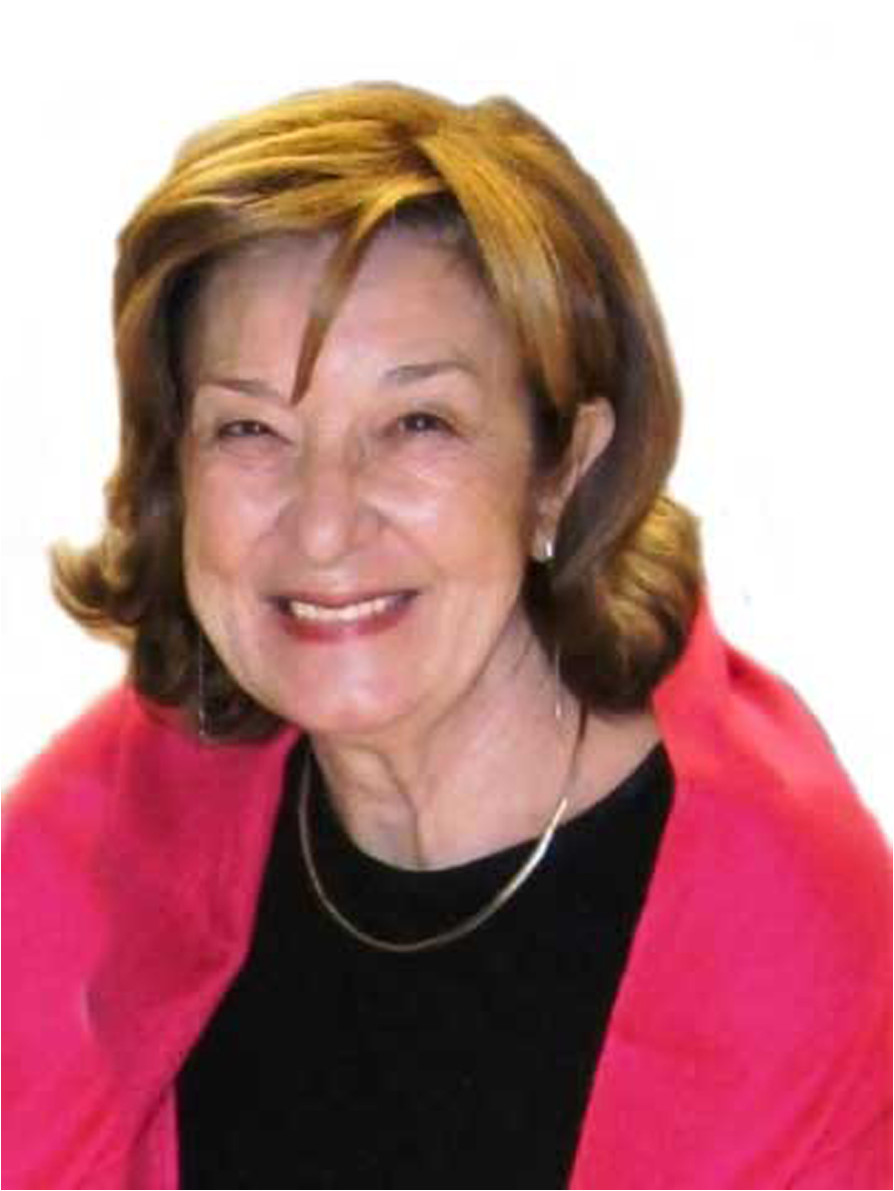
**Antonia Trichopoulou is Professor Emeritus at the School of Medicine, University of Athens, Greece and Vice President of the non-profit Hellenic Health Foundation.** Trichopoulou was the first to develop a Mediterranean diet score to measure adherence to this diet and facilitate the study of its health effects.

**Antonia Trichopoulou, (Figure**1**)**

In purely descriptive terms, the traditional Mediterranean diet is the dietary pattern prevailing among the people of the olive tree-growing areas of the Mediterranean basin before the mid-1960s, that is, before globalization made its influence on lifestyle, including diet. Key determinants of the traditional Mediterranean diet have been climate, flora and hardship, the latter discouraging import or consumption of expensive, at that time, red meat [1].

The traditional Mediterranean diet is characterized [2] by high consumption of vegetables, fruits and nuts, legumes, and unprocessed cereals; low consumption of meat and meat products; and low consumption of dairy products (with the exception of the long-preservable cheeses). Alcohol consumption was common in the traditional Mediterranean diet, but generally in moderation and in the form of wine and, as a rule, during meals- in the spirit of the ancient Greek word ‘symposium’. Total intake of lipids could be high (around or in excess of 40% of total energy intake, as in Greece), or moderate (around 30% of total energy intake, as in Italy) but, in all instances, the ratio of the beneficial monounsaturated to the non-beneficial saturated lipids is high, because of the high monounsaturated content of the liberally used olive oil. Finally, fish consumption has in the past been a function of the distance from the sea but has been, overall, at a moderate level.

In a somewhat reductionist approach, the traditional Mediterranean diet can be considered as a mainly, *but not dogmatically, exclusive* plant-based dietary pattern. Of note, olive oil is a plant product (in fact a fruit juice) and so is wine.

The traditional Mediterranean diet has entered the medical literature following publications by the legendary Ancel Keys and his colleagues of results from their ‘Seven Countries Study’, initiated in the late 1950s [3]. An important conclusion of this study, based largely on ecological evidence, was that low content of saturated lipids in the Mediterranean diet could explain the low incidence of coronary heart disease in Mediterranean countries, through the reduction of blood cholesterol, a recognized major risk factor for this disease (the distinction between high (HDL) and low (LDL) density lipoprotein cholesterol was not known at that time). Later work, however, has shown that the traditional Mediterranean diet is not simply, or mainly, a cholesterol-lowering diet, but has a range of beneficial health effects.

Two developments in the early 1990s have led to an explosion of interest in, and studies of the health effects of, the Mediterranean diet: (1) The recognition that high intake of carbohydrates, particularly simple carbohydrates, may not be beneficial to health because they constrain the levels of the ‘good’ HDL cholesterol and increase the metabolically undesirable glycemic load. This has shifted interest to innocuous, indeed beneficial, lipids, like those from olive oil [4]. Of note, carbohydrates and lipids are the principal sources of energy intake; at about 10% of total energy intake, proteins contribute less and with limited variability across individuals and populations in economically developed countries. (2) The operationalization of adherence (or conformity) to the traditional Mediterranean diet through a simple score, or variations of which, that have been used in a multitude of analytical (individual-based), rather than ecological observational, studies to evaluate the health effects of adherence to this diet [5]. It should be made clear that, in contrast to scores and diet pyramids developed in order to point to ‘optimal’ diets, the Mediterranean diet score is purely descriptive of the traditional Mediterranean diet. The fact that this diet has considerable beneficial health effects constitutes a ‘natural experiment’ that investigators try to understand and people benefit from.

Collectively, these studies have indicated convincing inverse associations with overall mortality [6] and with the incidence of coronary heart disease [7] and thrombotic stroke [8], compelling inverse associations with incidence of cancer overall [9, 10] (including, possibly, incidence of breast [11] and colorectal [12] cancer), likely inverse association with the incidence of adult-onset diabetes mellitus [13] and possibly with the incidence of hip fractures [14]. There have also been randomized trials supporting a beneficial role of the Mediterranean diet on the incidence of cardiovascular events [15] and of survival from coronary heart disease [16].

The traditional Mediterranean diet *can* be defined, however loosely, and has clearly beneficial health effects. Why it is health promoting, however, is not easy to answer. From the randomized trials, de Lorgeril infers that alpha-linolenic acid is a key factor [16], whereas the PREDIMED (Prevención con Dieta Mediterránea) primary prevention trial emphasizes extra virgin olive oil and nuts [15]. In an anatomy of the overall health effects of conformity to the Mediterranean diet in the Greek EPIC cohort (as reflected in the apparent reduction of total mortality), high consumption of plant foods accounted for 37.2% of the reduction (vegetables 16.2%, fruits and nuts 11.3%, legumes 9.7%), moderate alcohol intake, as contrasted to high or none for 23.5% of the reduction, whereas low meat intake accounted for 16.6% and olive oil (as reflected in the monounsaturated-to-saturated ratio) for 10.6%. The other components of the traditional Mediterranean diet score did not have a statistically significant impact, nor was there significant evidence for an over-additive synergism between any two components. The important role of olive oil in favoring high consumption of vegetables and legumes, however, could not be captured in the analysis [17].

As for mechanistic processes, the effect of alcohol on HDL, the high anti-oxidant content of this plant-based diet, the high content of fiber, and the low glycemic load of this high-lipid diet and other mechanisms have been considered but not adequately substantiated. Future studies may follow, or improve and enrich, our approach to disentangle the health effects of the components of the Mediterranean diet and of their mutual interactions [17]. They could also focus on the identification of the key compounds in this diet or biochemical or molecular mediators of its beneficial health effects. Meanwhile, people could try to adjust their diets to the principles of the traditional Mediterranean diet, as outlined above. After all, this diet is not only health promoting, as the overwhelming evidence indicates, but also delicious, as many of those who have tried variations of it readily acknowledged.

## The concept and operational definition of the Mediterranean diet

**Figure 2:**
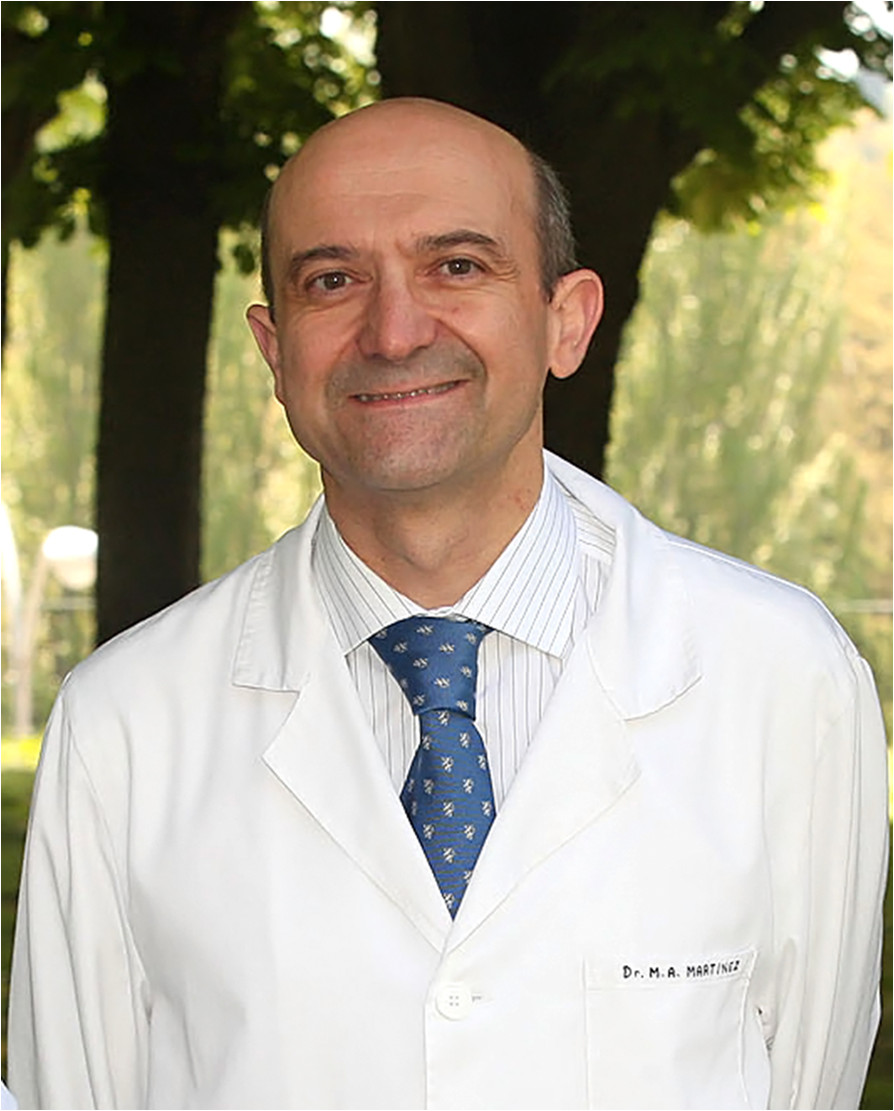
**Miguel A Martínez-González is Professor and Chair of the Department of Preventive Medicine and Public Health University of Navarra, Spain.** Martínez-González leads Network RD 06/0045 of the PREDIMED study, the first primary prevention trial to demonstrate that consuming a Mediterranean diet reduces the incidence of major cardiovascular events. He is also a principal investigator on the SUN (Seguimiento Universidad de Navarra) cohort study, and a visiting scholar at the Department of Nutrition, Harvard School of Public Health.

**Miguel A Martínez-González (Figure**2**)**

The concept ‘Mediterranean diet’ was developed to reflect the typical dietary habits followed during the early 1960s by inhabitants of the Mediterranean basin, mainly in Crete, much of the rest of Greece and Southern Italy [18]. It is essentially a frugal diet that was followed by poor rural societies [19].

More recently, the Mediterranean diet has been operationally defined in order to assess its role in analytical epidemiologic studies [20, 21]. The operational definition of Mediterranean diet most commonly used is the Mediterranean Dietary Score (MDS) proposed by Trichopoulou *et al*. in 1995 [5, 20] and updated thereafter [2]. The MDS is built by assigning a value of 0 or 1 to each of nine components with the use of the sex-specific median as the cut-off. For five beneficial components (vegetables, legumes, fruits + nuts, cereal and fish), persons whose consumption is below the sex-specific median of the sample are assigned a value of 0, and persons whose consumption is at or above the median are assigned a value of 1. A sixth beneficial component is the ratio of monounsaturated lipids to saturated lipids, in order to reflect the principal role of olive oil consumption in the traditional Mediterranean diet. A value of 1 is assigned to persons whose consumption is at or above the sample-specific median and a value of 0 is assigned to persons who are below the median. For components presumed to be detrimental (all meats, and all dairy products, which are rarely non-fat or low-fat in Mediterranean countries), persons whose consumption is below the median are assigned a value of 1, and persons whose consumption is at or above the median are assigned a value of 0. For alcohol, a value of 1 is assigned to men who consume between 10 and 50 g per day and to women who consume between 5 and 25 g per day. Thus, the total Mediterranean-diet score ranges from 0 (minimal adherence to the traditional Mediterranean diet) to 9 (maximal adherence) [2].

The MDS is based on sample medians and, therefore, its score is highly dependent on the specific characteristics of the sample. This fact may represent a limitation for the transferability of results to other samples. An alternative is to build scores according to absolute/normative cut-off points for the consumption of specific food groups (pre-defined servings/day or servings/week) [22–24]. This is the approach followed by the screener which was instrumental in performing the dietary intervention with the Mediterranean diet in the successful PREDIMED trial [15, 24, 25].

When compared with other ‘healthy’ diets, two elements of the Mediterranean diet are unique: 1) abundant fat intake is allowed provided that it comes from virgin olive oil, tree nuts and fatty fish, and 2) moderate intake of red wine during meals [17, 26]. Other components (fish instead of red meats, abundance of plant-based foods) are common with other ‘healthy’ diets. Alcohol should be included in the definition of the Mediterranean diet. The Mediterranean alcohol drinking pattern [26] seems a key element for reducing total mortality [17, 26].

The disparity of definitions for the Mediterranean diet may seem surprising. The reasons for the disparate definitions are diverse, complicated and not completely understood. Some historical reflections may shed light on the reasons explaining the different definitions. The Lyon Diet Heart Study was a landmark trial because it was the first randomized trial to show a strong cardiovascular protection for a dietary intervention using an overall dietary pattern. This trial included 605 patients who had had a previous myocardial infarction (that is, this was a ‘secondary’ prevention trial). These patients were randomly allocated to a so-called Mediterranean-type diet or a control diet following the guidelines of the American Heart Association Step I diet. The Mediterranean-type diet group received advice to increase the consumption of vegetables, fruits and fish, but to reduce the consumption of red meats. They were asked to replace butter and cream with a special linolenic acid-rich margarine. The results were impressive with a 73 percent relative reduction in the rate of coronary events after 27 months of follow-up [16]. The use of another type of fat different from olive oil might have opened the road to other modifications of the original definition of Mediterranean diet [27].

The most widely researched health benefits of the Mediterranean diet are the reduction in cardiovascular disease, including peripheral artery disease [15, 16, 27–29]. The available evidence to support a causal vascular protection is sufficiently strong with successful randomized trials [15, 16, 29]. Other benefits extensively researched include the prevention of type 2 diabetes [30, 31] and metabolic syndrome [32], cognitive impairment [33–35], and unipolar depression [36, 37]. The EPIC study has also provided some benefits against the occurrence of cancer [10]. The evidence of potential protection seems stronger for gastric, colorectal, and breast cancers, especially when alcohol is excluded from the definition [10, 38].

## The importance of redefining the Mediterranean diet in epidemiology

**Figure 3:**
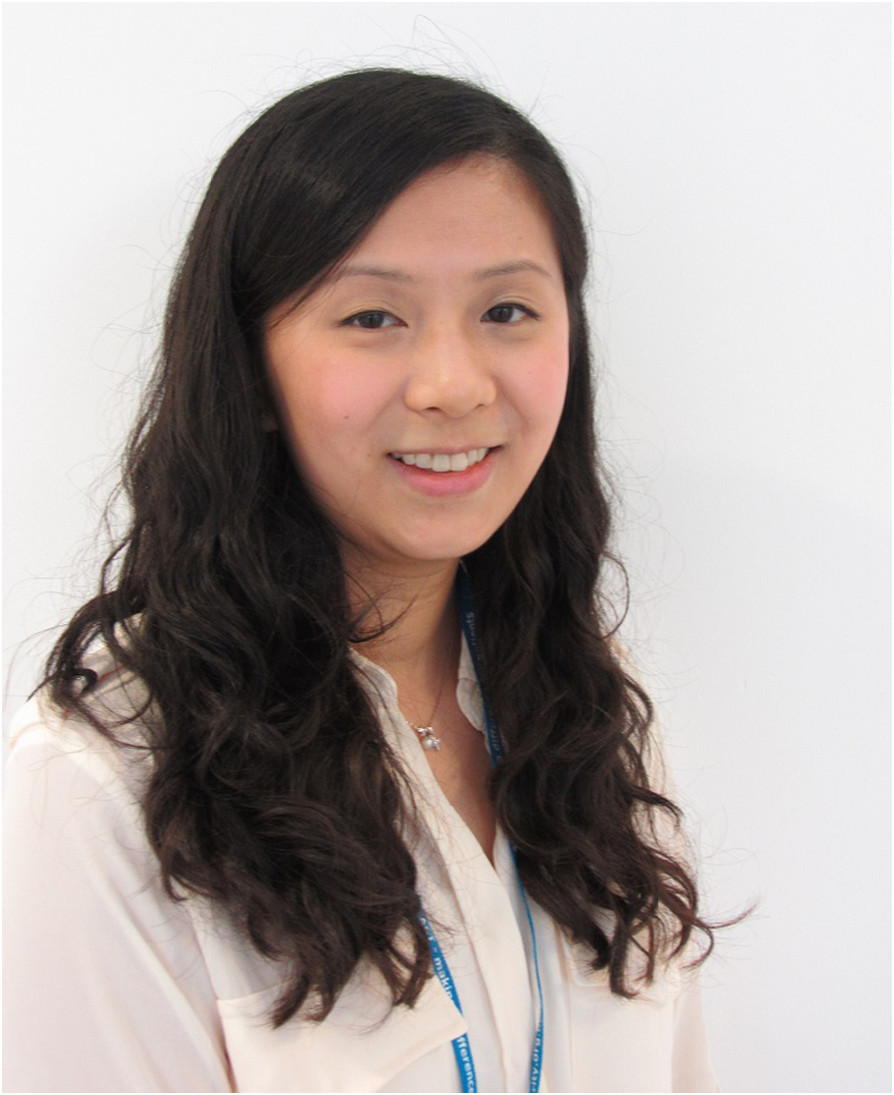
**Tammy Tong is undertaking her doctoral studies (PhD) at the MRC Epidemiology Unit in Cambridge, UK.** Her work is focused on assessing the applicability of the Mediterranean diet in the UK context, and in examining etiological associations of the diet with cardio-metabolic disorders.

**Figure 4:**
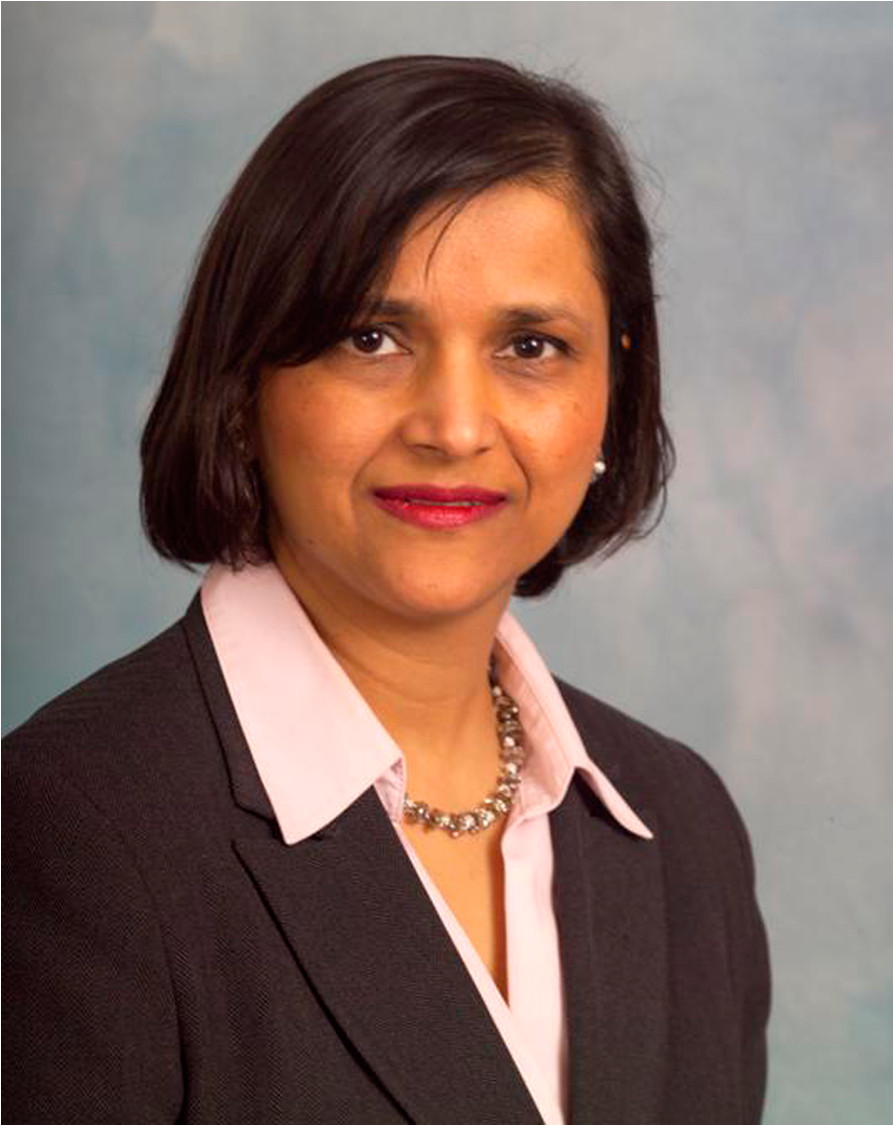
**Nita Forouhi is the Group Leader of the nutritional epidemiology program at the MRC Epidemiology Unit in Cambridge, UK.** Trained in Medicine, Epidemiology and Public Health, Nita is interested in etiology, prevention and between-population differences. Nita is leading a program of research that aims to understand the association between diet/nutrition and the risk of diabetes, obesity and related disorders. Her research has a particular focus on developing and using improved methods to assess diet, including the use of objectively measured nutritional biomarkers.

**Tammy YN Tong, Nita G Forouhi (Figures**3**and**4**)**

Mediterranean countries are historically among the healthiest countries in the world, recording relatively low rates of cardiovascular diseases and cancer as well as greater longevity. This ecological observation led to the idea of a healthy Mediterranean diet, based on traditional diets of regions such as Crete, other parts of Greece and Southern Italy [18, 19]. Offering a potential solution to improve health and well-being through reduction in chronic disease incidence and mortality, the ‘Mediterranean diet’ has been studied for its effects on a range of conditions in countries not limited to the original Mediterranean region.

Consistent with the findings of the landmark Lyon Diet Heart Study [16, 39] and the five-year PREDIMED trial [15, 31], a number of long-term observational studies supported protective roles of the Mediterranean diet against noncommunicable diseases [5, 34, 40–43]. The diet is also received favorably by the general population and government agencies alike, being rated joint third best diet overall by the US News & World Report [44], as well as being recommended by the UK National Health Service as a healthy meal choice [45]. A further ‘feather in the cap’ of the Mediterranean diet is its recognition by UNESCO as an intangible cultural heritage of several Mediterranean countries [46].

**Figure 5:**
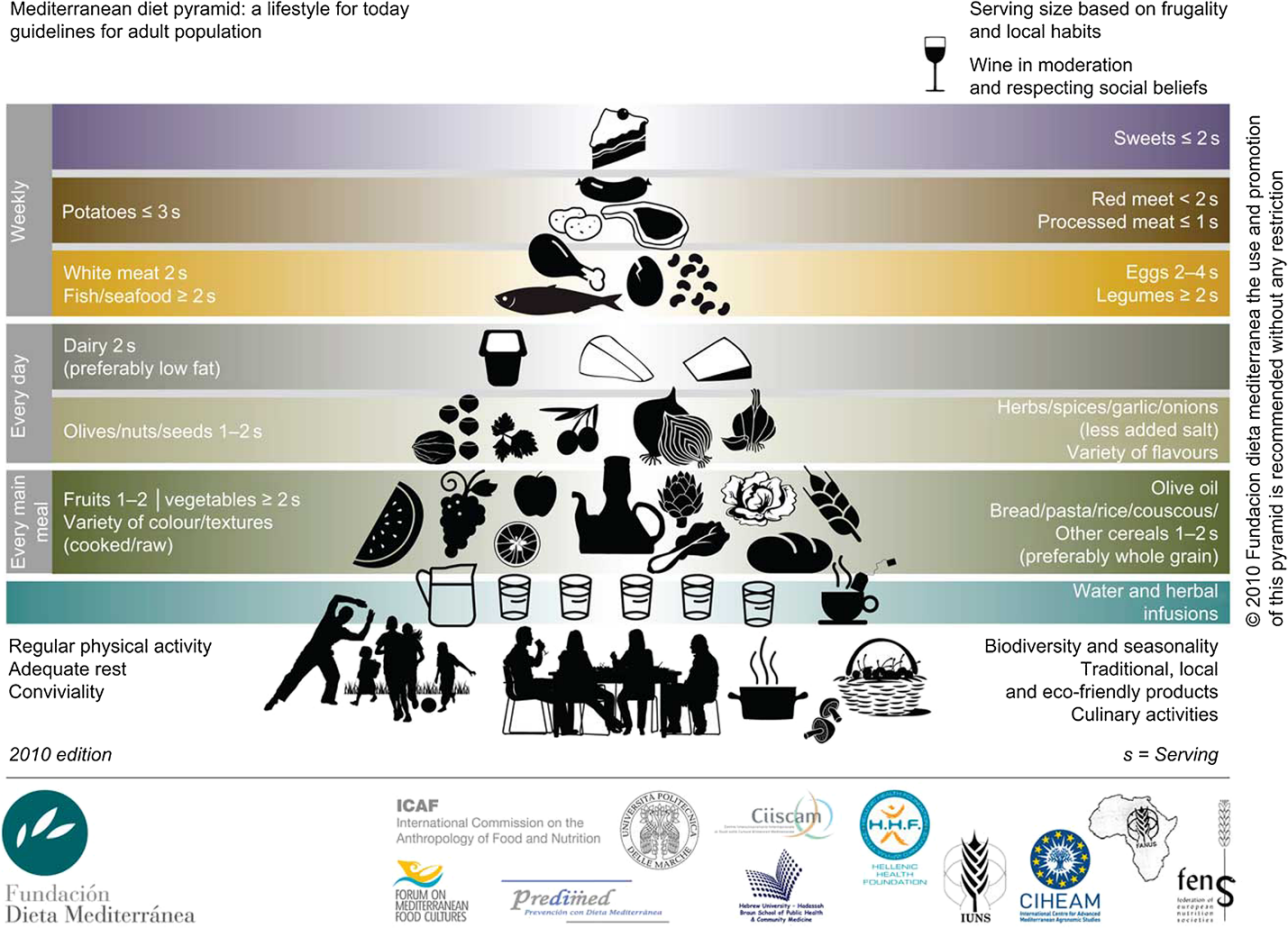
**The Mediterranean diet pyramid.** Reproduced from [19], who encourage use of this image without restriction*.*

The Mediterranean diet pyramid (Figure 5), as recommended by the Fundación Dieta Mediterránea, promotes a high consumption of cereals, fruits and vegetables; low consumption of red meats and sweets and moderate consumption of dairy, poultry and fish [18, 19]. Additionally, the diet also includes moderate consumption of wine and use of olive oil (replacing other forms of fats) as essential components of the diet. Both these factors can be considered reasonable recommendations, given past evidence of health benefits for cardiovascular health associated with olive oil [47, 48] and moderate alcohol consumption [49].

To improve the evidence for the health benefits of the Mediterranean diet, more systematic and quantitative approaches are needed in research practice. To date, applicability of the Mediterranean diet to non-Mediterranean countries has not been established. The premier study in Greece by Trichopoulou *et al*. [5] evaluated eight dietary factors as components of the Mediterranean diet: vegetables, legumes, fruits and nuts, grains, meats, dairy, alcohol, as well as dietary fats, with fish added later on as a ninth component [43]. However, while consumption of these factors provides a good approximation to a Mediterranean type diet under certain circumstances, it has several shortcomings. One problem is that the selection and use of the dietary information is too specific to the local populations studied. Therefore, when examining benefits of the Mediterranean diet in different populations, the patterns of consumption of key dietary components should be examined first in order to make appropriate adjustments.

Considering many advances in dietary research in the past decade, modifications to existing methods of assessing adherence to the Mediterranean diet are also warranted. This is particularly so since most studies have not evaluated the health benefits of adherence to the Mediterranean diet that was originally characterized in the Mediterranean region [18, 19]. For example, when assessing the Mediterranean diet, it still remains unclear as to whether, for alcohol intake, any distinction should be made between red wine and other types of alcohol, even though wine is the form of alcohol traditionally consumed in Mediterranean countries [18, 19, 50]. While some epidemiological studies have reported potential health benefits of moderate wine consumption, the extent of these health benefits seems to be less apparent for other alcoholic beverages [51, 52]. However, only a few studies on the Mediterranean diet recognized wine as a standalone component instead of total alcohol [53, 54]. Future observational studies should take into account this differentiation, and ideally incorporate wine only as an element of the Mediterranean diet when assessing adherence to this dietary pattern. It will be of particular interest to examine differences in association with disease risk when wine alone versus total alcohol intake is included.

Moreover, high intake of dairy products is considered as adverse in the landmark publications on the Mediterranean diet and health [5, 43]. However, recent epidemiological evidence suggests lower cardiometabolic risk associated with consumption of dairy products, in particular fermented dairy products [55–58]. Importantly, moderate amounts of fermented dairy products are also traditionally consumed in Mediterranean countries [18, 19]. Similarly, grains and meat products are of interest, in regards to whether whole grains and refined grains, or unprocessed red meats, processed meats, and poultry should be distinguished.

Existing studies of the Mediterranean diet have used varying definitions of the diet and found associations of adherence to the diet with different health outcomes. However, none of them has fully examined the traditional Mediterranean diet, reflecting the difficulty of attempting to use a simple definition to describe dietary behavior which is inherently complex. Future research should, therefore, aim to amalgamate existing definitions of the Mediterranean diet with up-to-date scientific evidence of health outcomes associated with individual components. Furthermore, the Mediterranean diet is essentially part of a lifestyle, requiring the simultaneous consideration of other non-dietary behavioral factors when assessing its effects. What the Mediterranean diet, therefore, means in the context of some countries with distinct cultural diets and lifestyles, such as for instance in China, India, and parts of Africa, needs further research and thought, despite the fair amount of evidence among the Western and, particularly, Mediterranean countries.

## Mediterranean diet: an Indian perspective

**Figure 6:**
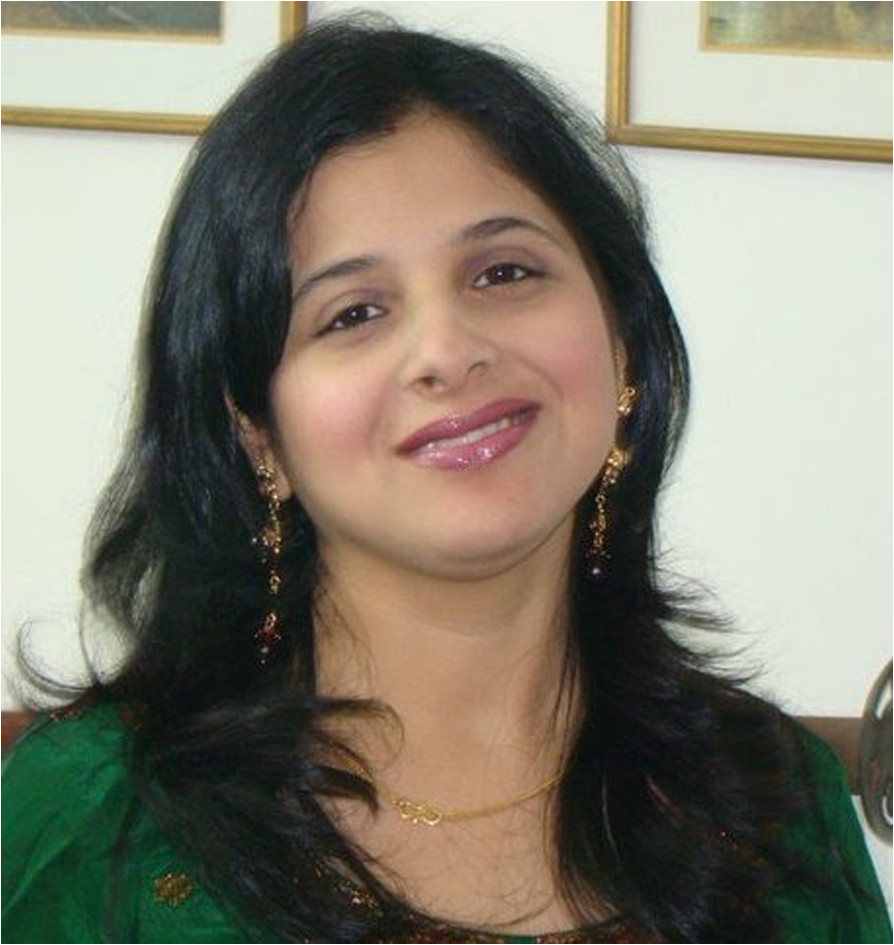
**Shweta Khandelwal is currently working as a Research Scientist and Adjunct Assistant Professor at the PHFI, New Delhi.** She is a trained public health nutritionist and her current research is focused on exploring the role of omega-3 fatty acids on cardiovascular disease risk factors among the Indian population. She is also the lead for capacity building initiatives in Public Health Nutrition at PHFI and CCDC.

**Figure 7:**
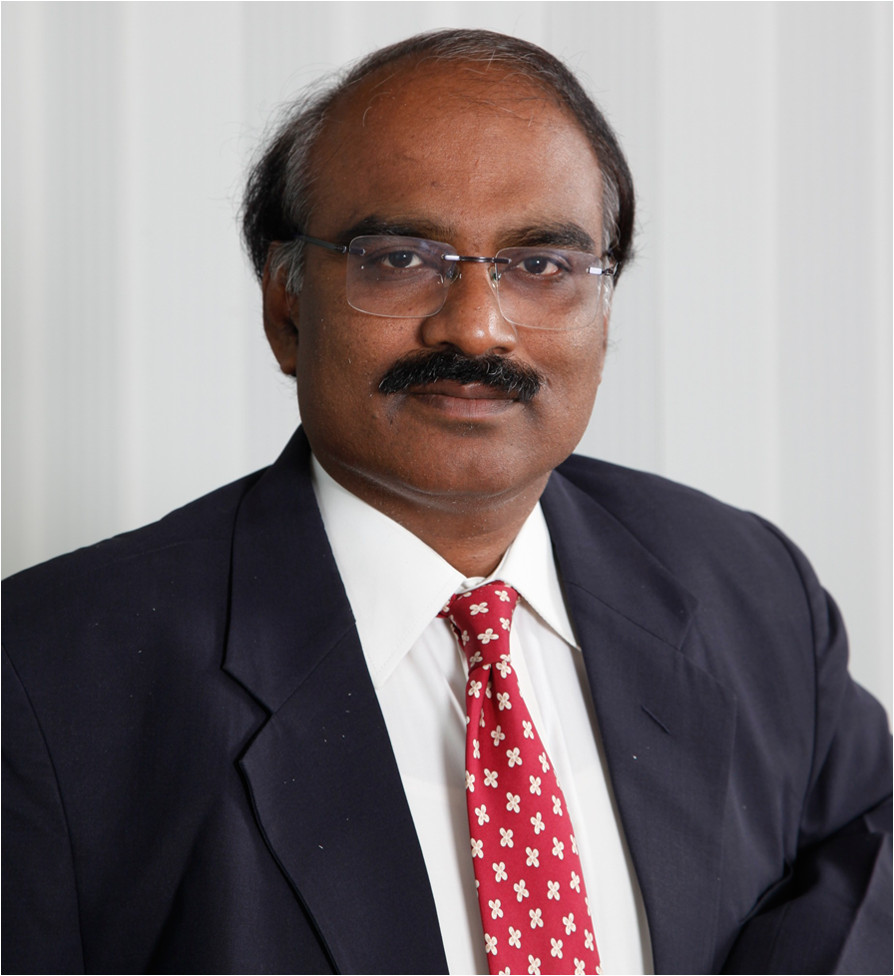
**Dorairaj Prabhakaran is a cardiologist and epidemiologist by training.** He served as Additional Professor of Cardiology at AIIMS until 2007, when he became the Executive Director of the Initiative for Cardiovascular Health Research in Developing Countries (IC Health) and the CCDC. He is also the Director of the NHLBI-United Health funded Center of Excellence in Cardio-metabolic Risk Reduction in South Asia (CoE-CARRS) at the PHFI which is one of the eleven centers worldwide supported under the Global Health Initiative of NHLBI, USA. His research work spans from mechanistic research to understand the causes of the increased propensity of cardiovascular diseases among Indians to developing potential solutions for CVDs through translational research and human resource development. CVDs, cardiovascular diseases; NHLBI, National Heart, Lung and Blood Institute.

**Shweta Khandelwal, Dorairaj Prabhakaran (Figures**6**and**7**)**

The term ‘Mediterranean Diet’ is usually employed to indicate the typical diet of almost 16 countries located on the Mediterranean seacoast [28, 59]. Several publications report the cardio-protective benefits conferred by this dietary pattern [15, 59–63]. However, the applicability and suitability of the Mediterranean diet in the Indian context have not been studied previously.

India is in the midst of a ‘nutrition transition’, where changes in diet parallel an expanding industrial economy and a rapidly progressing epidemic of obesity and non-communicable diseases, particularly in urban locations [64, 65]. Furthermore, it is well known that Indians have a higher risk of developing diabetes and cardiovascular disease (CVD) than other populations [66, 67]. Although the reasons for this are unclear, diet could play a major role. In this regard it is attractive to speculate that the Mediterranean diet may exert a protective role. Here, we discuss the constituents of the Indian diet that are similar to the Mediterranean diet, and evaluate the potential of adapting the Mediterranean diet to an Indian context.

By and large, a typical Indian diet is rich in carbohydrates (largely refined cereals), low quality proteins (largely from legumes), rich gravies (high in saturated fats and salt) and has low levels of fresh fruits and vegetables. The overall meat consumption is not very high, even among those who report non-vegetarian food consumption [68–70].

Some of the Mediterranean diet constituents and their suitability in the current Indian context are outlined in Table 1 and discussed below.

**Table 1:** **Summary of the Mediterranean and Indian diets**

Dietary components commonly consumed	Mediterranean diet	Indian diet	Evidence on health benefits of the Indian counterparts
Oils	Olive oil	Ground nut oil Mustard oil	Compared with persons consuming sunflower oil, those using mustard oil for cooking had an RR of 0.44 for IHD in the age-, sex-, and smoking-adjusted analysis. Similarly, persons using mustard oil for frying foods had a 71% lower risk (RR: 0.29; 95% CI: 0.13, 0.64) in multivariate analysis. When compared with all other fats and oils, the inverse association with mustard oil remained [70].
Alcohol	Wine	Beer and whisky	The INTERHEART study found that while alcohol protected people from heart attacks in the large sample population from 52 countries, it appeared to be harmful to Indians [71]. The Sentinel Surveillance cross-sectional study, analyzing data from 10 industrial sites in India, reported an odds ratio of 1.4 (1.0 to 1.9, *P* = 0.05) for CHD among alcohol users after adjusting for major confounders [72].
Proteins	More fish, sea foods, chicken and legumes. Less red meat	Most from legumes/pulses and less from non-vegetarian foods	Although fish consumption (among non-vegetarians) has been shown to improve lipid fractions among Indians and thus lower CVD risk [73], the consumption varies in different regions. Most Indians consume pulses much more frequently than fish [74–76]. In the Indian context, culture, traditions, customs and taboos influence meat consumption to a great extent, especially in the rural societies. However, there have been studies that show that urbanization has been causing a rise in demand for meat products. The per capita meat consumption in India is only around 44.39 gm/capita/day as compared to world consumption of 116.82 gm/capita/day.
Omega-3 fatty acids	Fish	Mustard oil, flax seeds	Mustard oil is the source of the short chain omega 3 fatty acids in Indian diets [77–79].
Carbohydrates	Whole grains, complex carbohydrates and more fiber	Refined cereals and processed foods	Evidence from some studies shows a positive association between refined carbohydrates and insulin resistance. Experiments with complex whole grains and fiber have yielded a better glycemic profile [80–82]. However, dietary data collection methods which are largely self-reported in these studies need to be standardized further for better quality data.
Dairy	Low consumption	Frequent use of dairy in beverages, desserts	Observational data suggests that dairy consumption in India was inversely associated with obesity. After controlling for potential confounders, the risk of being obese was lower among women (OR = 0.57; 95% CI: 0.43 to 0.76) and men (OR = 0.67; 95% CI: 0.51 to 0.87) who consume ≥1 portion of plain milk daily than those who do not consume any milk [83]. However, interventional studies are warranted to confirm this association.
Fruits and Vegetables	Fresh raw fruits and vegetables	Low consumption of fresh fruits and vegetables	The protective role of fruits and vegetables especially for better cardiovascular health (better lipid profiles, immunity, blood glucose levels and so on) has been ascertained in multiple studies globally but high costs, perishability and lack of awareness in some societies are challenging, especially in India [84–88]. Educational campaigns from school level coupled with policy interventions are needed to enhance consumption and improve heart-health.

CHD, coronary heart disease; CI, confidence interval; CVD, cardiovascular disease; IHD, ischemic heart disease; RR; relative risk.

In India, cooking oils vary considerably depending upon the region. However, some mono unsaturated fatty acid-rich oils in India similar to olive oil include ground nut oil, rice bran oil and mustard oil. There is not much evidence on the cardio-protective effects of oils used in Indian cooking. However, some studies suggest that mustard oil conferred about 50% lower risk reduction for ischemic heart disease among the Indian population. Even rice bran oil has been shown to have hypolipidemic effects [71, 78, 89]. Further evidence on long term usage of these oils on cardiovascular health from good quality longitudinal studies is warranted. Olive oil has not gained huge popularity in India until now as a result of its cost, as well as its unsuitability for Indian frying conditions. However, recent subsidies provided by the Agricultural Ministry for olive cultivation confirm the increasing interest and the rising demand among Indians for olive oil [90, 91].

High consumption of fresh fruit and vegetables is a principal characteristic of the Mediterranean diet. Although India is the second largest producer of fruits and vegetables in the world (annual production of 94 million tons), the consumption per capita is quite low and has steadily declined in the last 50 years (120 to 140 g/day) [92]. A number of studies have reported a declining fruit and vegetable consumption pattern in different Indian populations [68, 84, 87, 88]. The most documented reasons for sub-optimal consumption involve affordability, awareness and access issues [93]. India can learn from some of the successful strategies to increase consumption in other countries [92, 94]. Most of the evidence supports starting early and using multi-component interventions for increasing fruit and vegetable intake [95, 96]. Inexpensive, culturally-acceptable and feasible interventions for boosting the fruit and vegetable consumption must be piloted and scaled up if successful. Policy interventions, such as subsidies on growing and storing fruits and vegetables, can offer sustainable solutions for enhancing consumption among developing countries such as India [97].

Key to the Mediterranean diet, consumption of legumes may be associated with a reduced risk of coronary heart disease (CHD) [98, 99]. Legumes are high in bean protein and water-soluble fiber, and are a good source of proteins, vitamins, minerals, omega-3 fatty acids and non-starch polysaccharides [77]. Per capita availability of legumes in India has decreased from 60 g in 1950 to 38 g in 1990, a reduction of nearly 40 per cent [100]. On the other hand, the per capita availability of cereal and millets has increased from 330 g to 470 g in spite of a four-fold increase in population. The cereal-to-pulse ratio, which should be ideally 8:1, has risen from 6:1 to 12:1 [99]. Even though pulses production increased by 3.35% per year during the last decade, the cost of production and consequent prices are too high to be affordable to many people; to increase production at lower cost is a bigger challenge. Experts suggest that technological efforts need to be supported by the right policy environment to leverage research and development in agriculture [101].

Another important item in the Mediterranean diet is fish, which owes its heart-healthy attribute largely to the long chain omega 3 fatty acids (n-3) [102]. While fish is widely consumed in the Mediterranean diet, consumption in India varies considerably depending on the region. Studies indicate that irrespective of the fish eating behavior, the plasma and erythrocyte levels of n-3 are usually very low across the Indian population [103, 104]. This may be because the consumption of n-3 rich foods is not frequent and when subjected to intense cooking methods, even the small available amounts get nearly eliminated. Several studies from other parts of the world have also looked at supplementation with n-3 as an isolated nutrient versus whole fish consumption [105]. The latter seemed to offer better cardiovascular health benefits. This may be because of additional protective constituents (such as fiber, protein, minerals and so on) or their synergistic effect in fatty fish as a whole.

Indian diets also have some alternative sources of n-3, such as mustard oil, some nuts and flaxseeds [106, 107]. However, these sources usually contain the shorter chain n-3, which need to get converted *in vivo* to their longer chain counterparts to offer a similar cardio-protective role. This conversion (dependent on the elongase and desaturase enzymes) is usually limited due to an excess of omega-6 fats (which compete for the same enzymes) in Indian diets [108]. However, a few studies in India have shown a modest beneficial impact especially on lipid profiles of adults when their diets were supplemented with flaxseeds and mustard oil [109, 110].

In terms of whole grains, Indian diets are rapidly transitioning. The traditional home cooked meals consisting largely of coarse grains and whole cereals are now replaced by cheaper refined versions. The latter are devoid of the fiber and other healthier components of complex carbohydrates. Recent studies in India have established strong positive associations between refined grain intake and type 2 diabetes, and confirm the protective effect of fiber, which is contained in whole grains [80–82]. Carbohydrates are integral to Asian Indian dietary traditions and re-introduction of culturally acceptable, traditional, carbohydrate-rich grains with high nutrient density may be a prudent step in reducing disease burden in this population.

While moderate wine consumption is typical in those consuming a Mediterranean diet, Indians are usually characterized as binge drinkers, largely consuming whisky or beer, in contrast to everyday wine consumers from western and European countries. The pattern of consumption also varies; in India people usually consume alcohol before meals while in other countries, it is consumed along with meals. The impact of alcohol consumption on CVD risk in India has been described in two studies (Table 1). The differential preference in the type of alcohol and pattern of drinking seem to reverse the cardio-protective effect conferred by small-moderate quantities of everyday wine consumption in other populations. Longitudinal data evaluating the role of alcohol in CVD risk among Indians are currently unavailable but urgently warranted.

Processed red meat is associated with a higher CVD risk profile [111, 112]. While red meat consumption is generally low in those adopting a Mediterranean dietary pattern, the UN Food and Agriculture Organization (FAO, 2007) reported Indians' per capita annual consumption of meat is rising [113]. Although the consumption statistics are still lower than the global average (Indian per capita annual consumption is about 5 to 5.5 kilograms or 11 to 12 pounds; and for the rest of the world, it is about 38 kilograms or 83.7 pounds), the steady rise in meat consumption among Indians reflects changing dietary preferences. Religion, and to some extent income, dominates the meat consumption pattern in India. While Hindus avoid beef, Muslims shun pork among the non-vegetarian populations in India. Longitudinal data from studies assessing the association between red meat consumption in India and CVD outcomes are needed.

The emphasized need for a higher quantity and quality of nutrition studies becomes even more relevant because nutrition research in India is still very nascent. Poor emphasis on and lack of academic/professional training in nutrition epidemiology in developing countries constraints the public health researchers and often yields sub-optimal data quality [114, 115]. Further, the commonly employed dietary data collection methods in Indian studies are not well standardized and contain self-reported information. These limitations further prevent high quality evidence building in the field of nutrition research.

Indians are already known to have higher cardiovascular disease risk than other populations [66, 116, 117]. Since unhealthy diet exacerbates the already high cardiovascular risk profile, well-designed nutritional epidemiological studies are warranted in the Indian population. Successful dietary interventions need to be adapted, particularly for dietary patterns rather than isolated nutrients, and tested in Indian settings for comparison with available global evidence. The role of individual constituents of the Mediterranean diet, their interactions with each other and with other items consumed concomitantly, along with various types of processing in traditional Indian mixed dishes, may alter some of their preventive properties and may also contribute substantially to increased CVD risk [118]. High quality intervention studies, such as the PREDIMED trial [15], assessing the acceptability of the Mediterranean diet or comparable constituents and their effect on the risk of major cardiovascular events in India are warranted. Until such data are available, Indians should be encouraged to consume a scientifically proven and contextually acceptable healthy dietary pattern comprising whole grains, fresh fruits and vegetables, good quality proteins (from pulses, chicken or fish) and some dairy products. Additionally, resources need to be urgently invested in strengthening nutrition research infrastructure and training to conduct and analyze high quality intervention and longitudinal studies in India. Strategies promoting collaborative studies and opportunities to build capacity in public health research should be deeply encouraged.

## Reflections on definitions and health benefits of the Mediterranean diet

**Figure 8:**
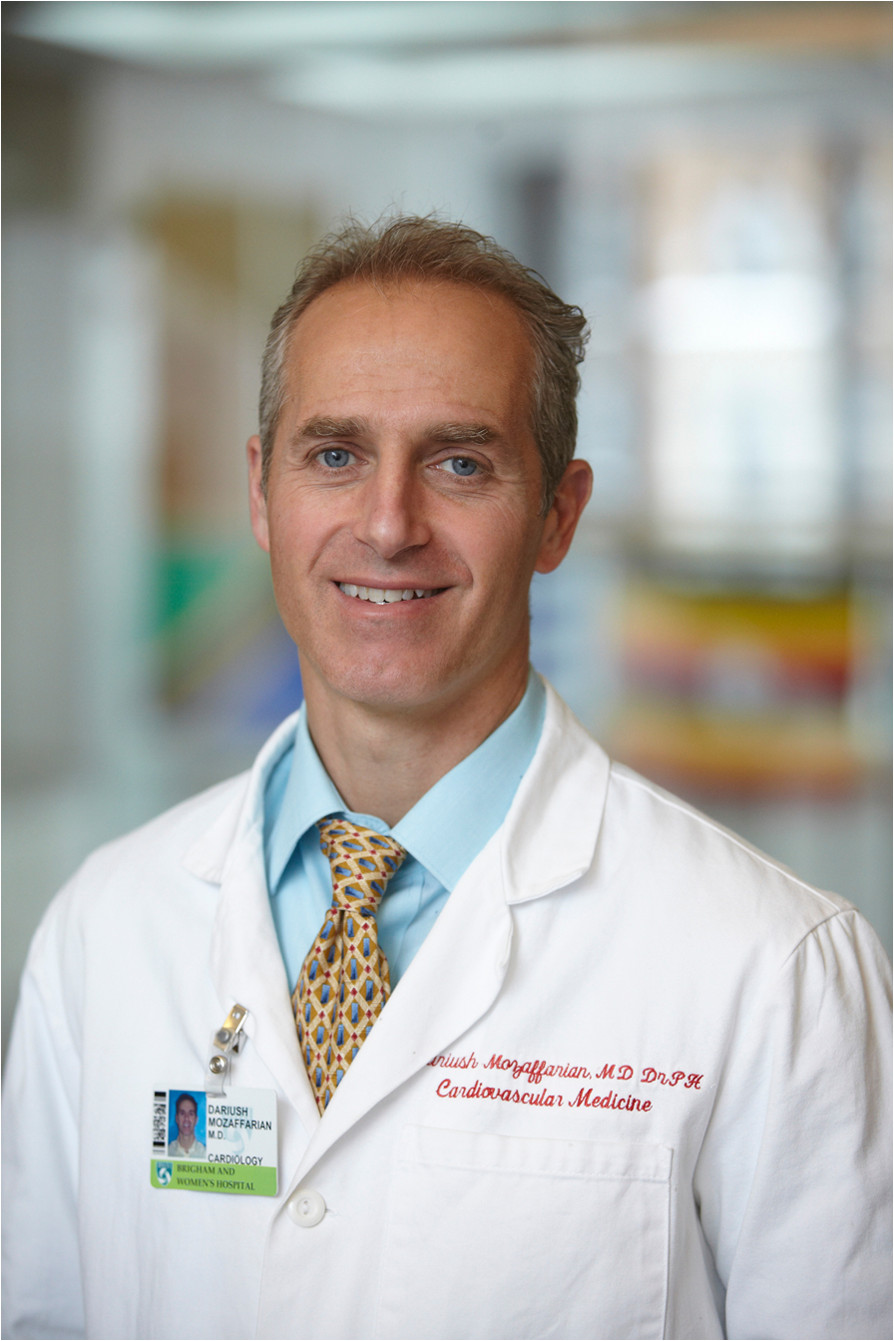
**Dariush Mozaffarian is Dean of the Friedman School of Nutrition Science & Policy at Tufts University.** His research focuses on the effects of lifestyle, particularly diet, on cardiometabolic health and disease, on the global impacts of suboptimal nutrition on chronic diseases, and on the effectiveness of policies to improve diet and reduce disease risk. Image credit: Kent Dayton.

**Dariush Mozaffarian (Figure**8**)**

An impressive and ever-expanding body of evidence has taught us that overall dietary quality strongly influences health, in particular risk of cardiometabolic diseases such as coronary heart disease, diabetes, and obesity [119]. Indeed, suboptimal diet quality is now the leading modifiable cause of death and disability in the world [120]. In contrast to the erroneous notions that diet quantity – how much a person eats – or isolated single nutrients are most important, the most relevant characteristics of healthful diets are the overall patterns of foods consumed.

Among various dietary patterns, consistent and compelling evidence indicates that traditional Mediterranean-style diets produce substantial health benefits. Diverse cultures and agricultural patterns exist in the Mediterranean region: there is no one, pure ‘Mediterranean diet’. Still, as discussed in the previous sections, traditional Mediterranean diets share fundamental characteristics, which either individually or together have been proven to improve cardiometabolic health. Because of this abundance of beneficial foods, such diets are also naturally lower in harmful foods such as highly processed snacks, cereals, and similar ready-made products; red and processed meats; and other refined grains, starches, and sugars [121].

Ecologic comparisons, prospective cohort studies, and randomized trials consistently demonstrate significant beneficial effects of Mediterranean-type diets and their components on cardiometabolic risk factors and disease endpoints [15, 119, 121–123]. The Spanish PREDIMED trial demonstrated a reduction in the risk of cardiovascular events by approximately 30% when participants were advised to follow a Mediterranean diet, supplemented with either nuts or extra-virgin olive oil [15]. Notably, extra-virgin olive oil largely replaced regular (non-virgin) olive oil, suggesting that the benefits of olive oil may be more closely related to bioactive compounds in extra-virgin oils [124] rather than to monounsaturated fats *per se*. Mediterranean diets also improve glycemic control [125] and reduce the incidence of type 2 diabetes [31]. The key components of Mediterranean diets are also beneficial for weight loss in obese patients [126] and for preventing long-term weight gain in non-obese populations [127]. Thus, rather than focusing on reductions in total calories or portion sizes, or on increasing or decreasing isolated nutrients, an emphasis on overall diet quality according to types of foods consumed has the strongest evidence-base for reducing adiposity and preventing diabetes and cardiovascular diseases. The main exceptions to this food-focused approach may be dietary additives such as sodium and trans fat, because very similar foods can be consumed that are either higher or lower in these additives, indicating a separate need to target these nutrients.

How does the Mediterranean diet compare to other healthful diet patterns? One close relative is the Dietary Approaches to Stop Hypertension (DASH) diet, which shares many of the same characteristics. Notably, while the original DASH diet was lower in fat and higher in carbohydrate, controlled clinical trials demonstrate that a higher-fat DASH diet, rich in healthful vegetable oils and nuts, produces even larger cardiometabolic benefits than the original low-fat DASH diet [128, 129]. People are also increasingly asking about vegetarian or vegan diets to improve their health. Unfortunately, because such diets are defined only by what is *not* consumed, the concept provides little accurate guidance for health. For instance, French fries, soda, and ketchup are vegetarian, as are refined grains, sugars, starches, sodium, and industrial trans fat. It is true that people who choose to be vegetarians or vegans are often health-conscious, so that they more often select healthier, minimally processed foods consistent with a Mediterranean diet [130]. However, a vegetarian or vegan diet *per se* – that is, the sole absence of animal products –has little influence on health, as true healthful diets are best defined by what *is* consumed, while also being characterized by lower consumption of unhealthful foods, many of which are actually ‘vegetarian’.

Unfortunately, diets in the Mediterranean region have worsened over time. In Crete, a Mediterranean island with historically low rates of chronic disease, the diets now contain less fruit and olive oil and more meats than diets of earlier generations, with associated population increases in serum cholesterol and adiposity [131]. A global dietary Renaissance is required, returning the traditional Mediterranean diet to its primacy in the region and, crucially, incorporating our knowledge of its numerous health benefits to practical, regionally tailored dietary guidance and policies worldwide.

## Mediterranean diet: from tradition and empiric description to modern science

**Figure 9:**
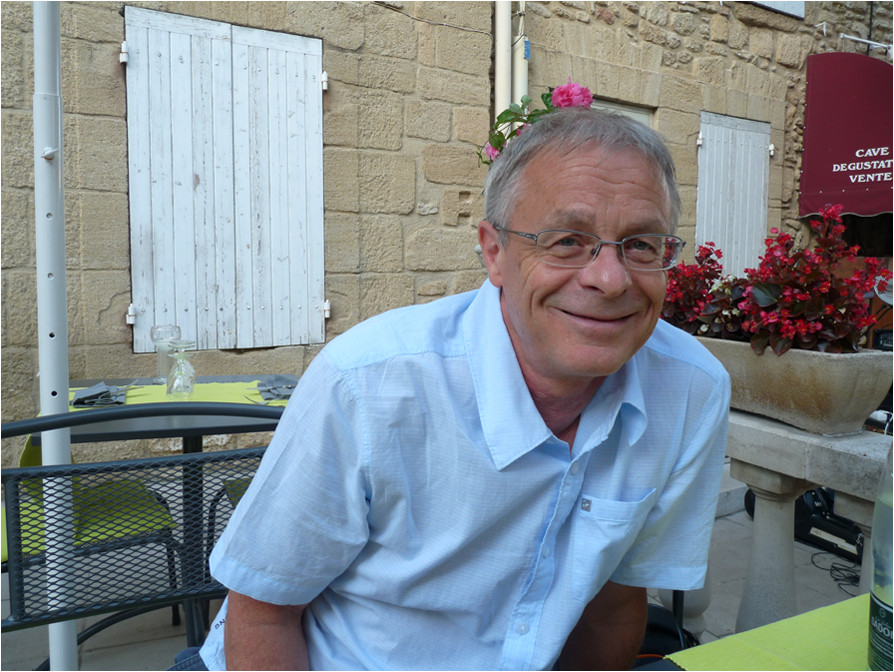
**Michel de Lorgeril is a cardiologist and nutritionist at the French National Centre for Scientific Research and the School of Medicine at Grenoble University, France.** In the 1990s he proposed a theory to explain the French paradox (low mortality rate from cardiac disease in France compared with UK and USA despite similar risk profiles), and his research group demonstrated that the plant omega-3 fatty acid (alpha-linolenic acid) is cardioprotective. Michel de Lorgeril was the principal investigator on the landmark Lyon Diet Heart Study, the first clinical trial to demonstrate the beneficial effects of the Mediterranean diet in the prevention of ischemic heart disease.

**Michel de Lorgeril (Figure**9**)**

The term ‘*Mediterranean diet*’ usually describes the dietary habits of populations living near the Mediterranean Sea [27]. The definition of the Mediterranean diet varies with geography, historical time and the nationality of the authors. In reality, the traditional dietary habits of the Greeks in 1950 were neither those of the Italians at that time, nor those of the Spaniards or Lebanese in 2014, although all of them do live on the shores of the Mediterranean Sea. These differences mainly explain the controversy about the definition of the Mediterranean diet.

After years of biological and medical research [27], it is definitely possible to look at the Mediterranean diet as a robust and complex **scientific concept**. It can be used by any practitioner, provided it is adapted to each specific geographic area and population, and called the *modernized* Mediterranean diet [27]. The next paragraphs will try to explain the shift from the empiric description of the traditional dietary habits of various Mediterranean populations to modern scientific medicine.

One good example is the *dietary fat issue*. It cannot be summarized with a single statement about olive oil. Briefly, Mediterranean people use several types of fats, from both plant and animal (including marine) sources. Many different fatty acids make up these fats. As shown in Table 2, comparing the *modernized* Mediterranean diet with a Western-type diet – grossly defined as the dietary habits of the US and North European (Finland, the Netherlands) populations investigated in the Seven Countries Study [3] –, it is important to differentiate oleic acid (the main monounsaturated fatty acid) provided by olive oil and the same chemical provided by animal fat. Oleic acid is indeed one of the main fatty acids of beef and pork fat. When the relations between the intake of oleic acid and any health item are analyzed within a Western cohort, investigators mainly analyze the relations with beef and pork consumption. When they do the same within a Mediterranean cohort, they analyze the relations with olive oil and the results are totally different. This may explain why certain (Western) experts refuse to acknowledge any health benefit from consuming olive oil, as if olive oil and oleic acid are the same things.

**Table 2:** **Dietary fats in the** ***modernized*** **Mediterranean diet compared with a Western-type diet**

Type of fat	Amount in the modernized Mediterranean diet compared with a Western-type diet	Key references
Total fat	slightly higher or not different	[16, 39]
Plant and animal saturated fats	much lower	[16, 39]
Plant monounsaturated fats	much higher	[16, 39]
Animal monounsaturated fats	lower	[27] and cited references
Animal n-6 polyunsaturated	lower	[27]
Plant n-6 polyunsaturated	much lower	[16, 39]
Plant n-3 polyunsaturated	much higher	[16, 39]
Animal (including marine) n-3 polyunsaturated	moderately higher	[16, 39]
Industrial trans fatty acids	much lower	[27] and cited references
Natural (ruminant) trans fatty acids	slightly higher or not different	[27] and cited references

On the other hand, while the *modernized* Mediterranean diet is not a vegetarian diet, it is definitely a plant-based diet. It is, therefore, crucial to identify the main sources of the *essential* omega-3 and omega-6 polyunsaturated fatty acids. Since olive oil is poor in both omega-6 and omega-3 fatty acids, what are the true sources of omega-3 and omega-6 fatty acids in either the traditional or the *modernized* Mediterranean diet? Along the same line, it is crucial to differentiate the main sources of the specific omega-3 fatty acids – those provided by plants and those provided by marine or terrestrial animals – and also the main sources of omega-6 fatty acids from either plants or animals (Table 2).

Finally, in the contemporary world where industrial foods are consumed by more and more people, it would be a mistake to still think that most saturated fats come from animal foods. Actually, saturated fatty acids also come from plants, such as the palm oil and cocoa butter incorporated in industrial foods. In the same way, it is essential to differentiate the (toxic) trans fatty acids produced by the industrial hydrogenation process and the (healthy) trans fatty acids naturally produced by ruminants and found in the dairy products typical of the Mediterranean diet.

All of these fat items, as well as other dietary items, illustrate how the empirical description of the traditional Mediterranean diet has become a modern scientific concept [27]. This is important to understand in order to design the optimal nutrition strategy to prevent disease. For instance, when testing the effects of the Mediterranean diet against cardiovascular complications in a controlled trial among French patients whose dietary habits were very different from the traditional Mediterranean diet, we were able to reproduce the main dietary aspects of the Mediterranean diet as regards fat (Table 2), without exclusively using olive oil [16, 39]. By advising our patients to use canola oil and canola oil-based margarine, plus some other Mediterranean foods – including olive oil, fatty fish, and nuts – we did reproduce the blood fatty acid profile characteristic of Mediterranean populations, with the appropriate omega-3/omega-6 ratio [132]. This may, at least in part, explain the impressive protection observed in the Lyon Diet Heart Study [16, 39], which was recently confirmed in the PREDIMED trial [15].

Thus, future trials testing the effects of a modern version of the Mediterranean diet in various clinical contexts (prevention of cancer or Alzheimer-type dementia) or future epidemiological studies should include that new knowledge in their protocols and designs. As an example, it will be important to differentiate the different *essential* (both omega-3 and omega-6) polyunsaturated fatty acids and also their food sources, animal versus plant (Table 2).

Finally, it is noteworthy that wheat, both whole and refined, is a major ingredient of the Mediterranean diet, mainly under the form of bread, but also of other typical Mediterranean diet foods, such as pasta and couscous [27, 133]. The physicians and nutritionists who are aware of the basic principles of the modernized Mediterranean diet recommend eating complex carbohydrates and whole grains, in particular bread and other wheat-based foods. However, the last decades have seen great changes in the prevalence and clinical presentation of two diseases linked to wheat: the celiac gluten-induced enteropathy and non-celiac gluten sensitivity [134, 135]. These changes have taken place as new wheat hybrids were introduced into human foods [134]. This is definitely a critical medical and environmental issue, which needs to be appropriately managed by physicians when their patients report new gastrointestinal or non-gastrointestinal symptoms after adhering to the modernized Mediterranean diet. The worst thing to do would be to deny the reality of these symptoms. There are alternatives to gluten-rich grains, and physicians and nutritionists should be careful to select such alternatives so as to respect the basic principles of the modernized Mediterranean diet. Thus, the gluten/wheat issue illustrates how a dietary pattern is not a static thing, but rather an ongoing change

In summary, even if wheat bread and olive oil are the very symbols of the *traditional* Mediterranean diet, a *modernized* Mediterranean diet concept makes it possible to obtain all the health benefits of typically Mediterranean dietary habits without olive oil or wheat bread. In other words, the *modernized* Mediterranean diet concept opens the way to a scientifically-founded protective dietary pattern which could be independent from the Mediterranean geography, climate and cultures. Future research – for instance when constructing a modern Mediterranean diet score in observational epidemiologic study – will have to integrate that new knowledge [134, 135].

## Authors’ original submitted files for images

Below are the links to the authors’ original submitted files for images. Authors’ original file for figure 1 Authors’ original file for figure 2 Authors’ original file for figure 3 Authors’ original file for figure 4 Authors’ original file for figure 5 Authors’ original file for figure 6 Authors’ original file for figure 7 Authors’ original file for figure 8 Authors’ original file for figure 9 Authors’ original file for figure 10
