# Supplementary figures and images for: Definitions and potential health benefits of the Mediterranean diet: views from experts around the world
Source: BMC Med. 2014 Jul 24;12:112. doi: 10.1186/1741-7015-12-112 (PMC4222885; doi:10.1186/1741-7015-12-112)

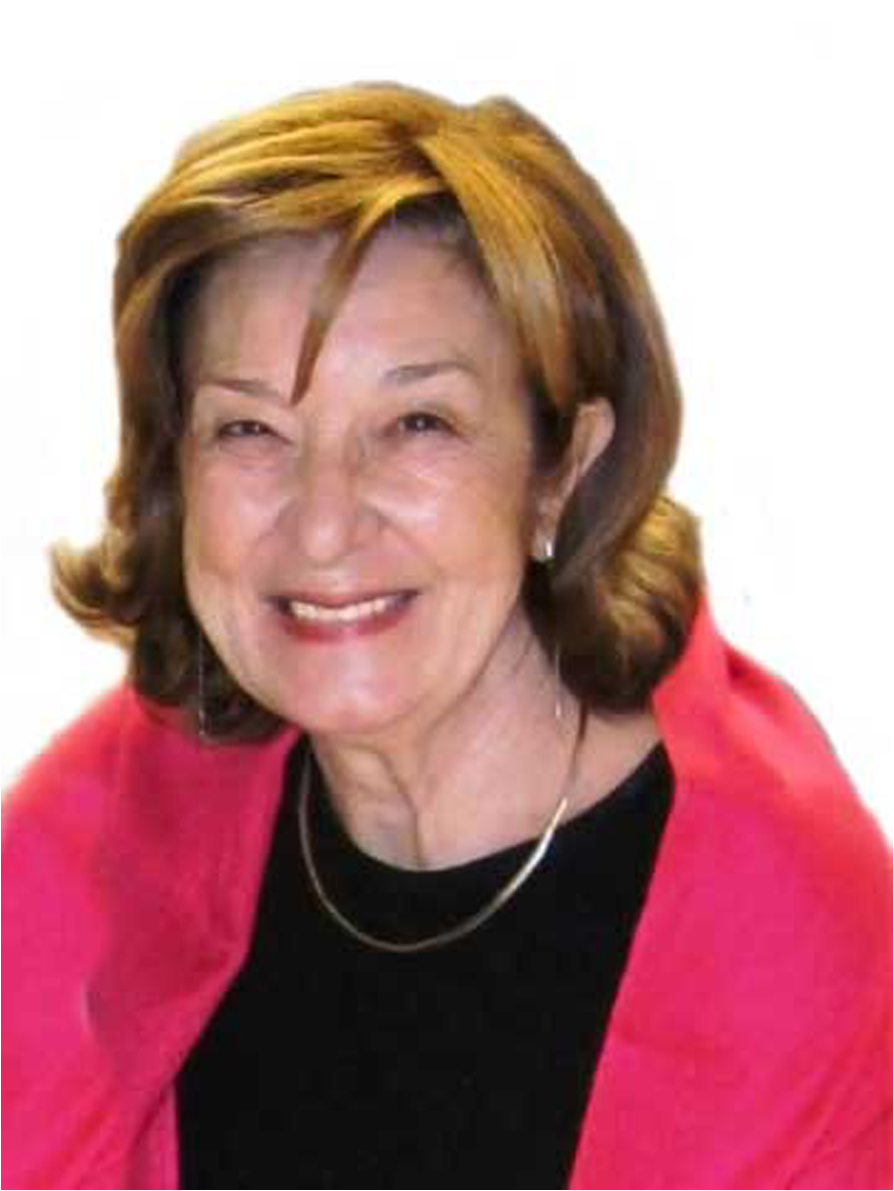

Supplement: Supplementary file 1 — Authors’ original file for figure 1 [file 12916_2014_994_MOESM1_ESM.tiff]

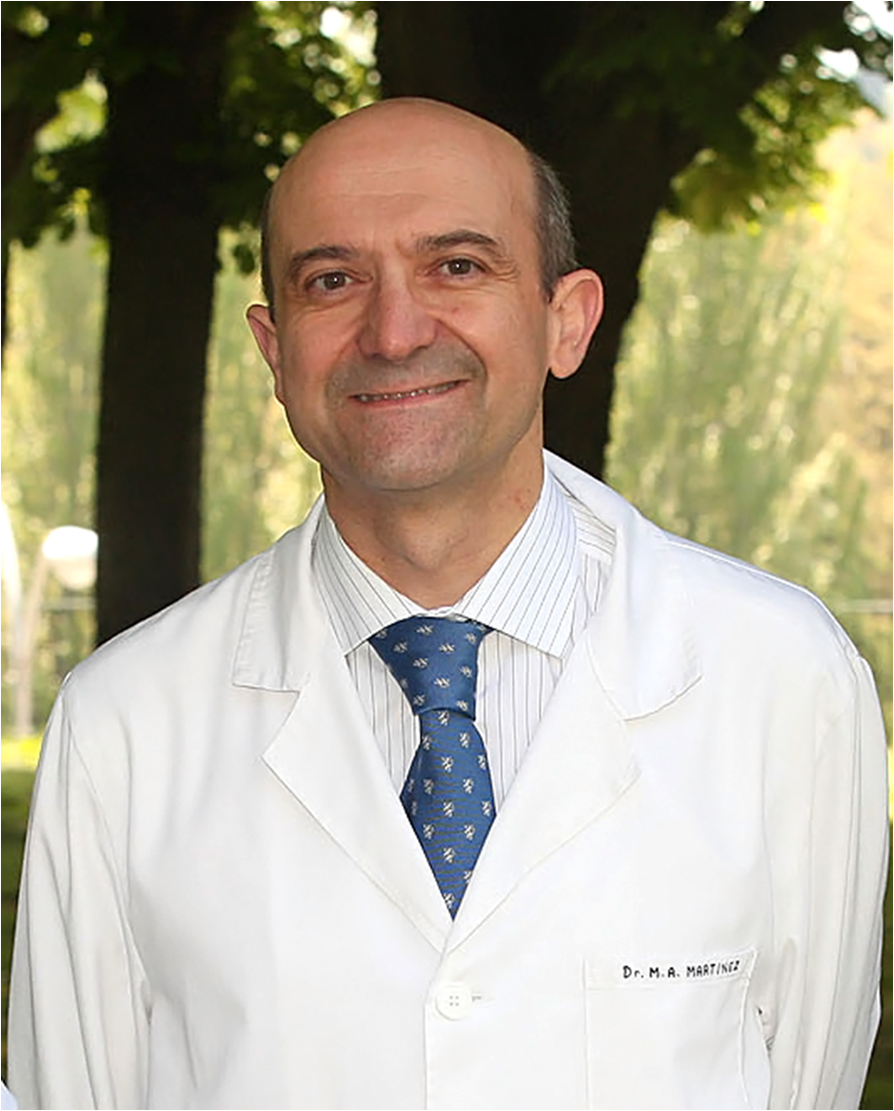

Supplement: Supplementary file 2 — Authors’ original file for figure 2 [file 12916_2014_994_MOESM2_ESM.tiff]

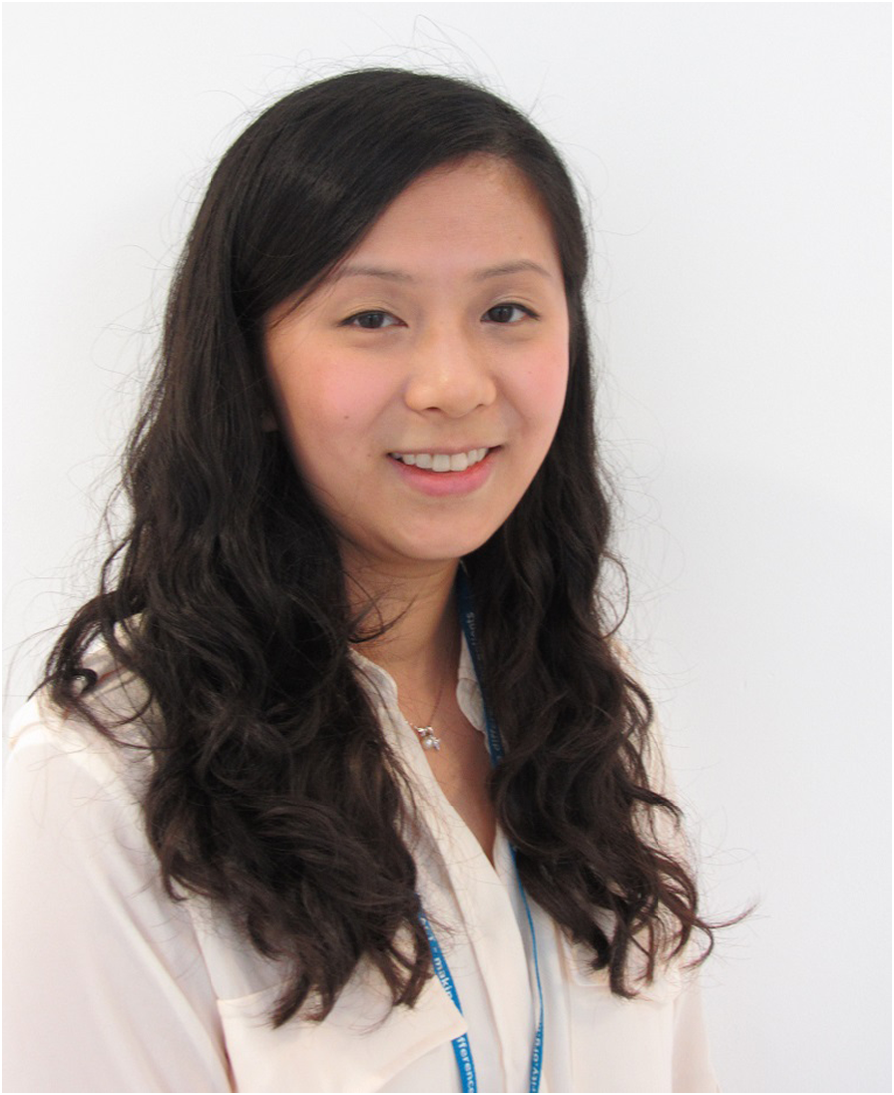

Supplement: Supplementary file 3 — Authors’ original file for figure 3 [file 12916_2014_994_MOESM3_ESM.tiff]

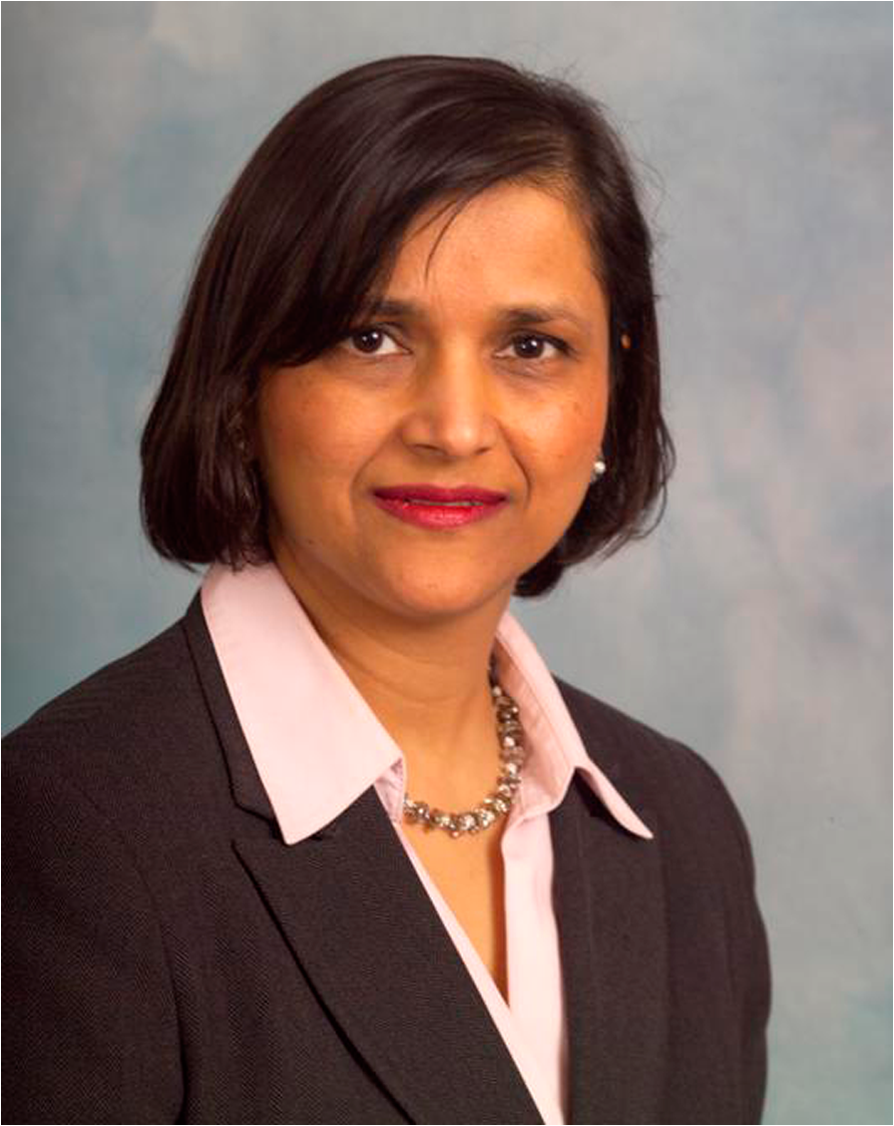

Supplement: Supplementary file 4 — Authors’ original file for figure 4 [file 12916_2014_994_MOESM4_ESM.tiff]

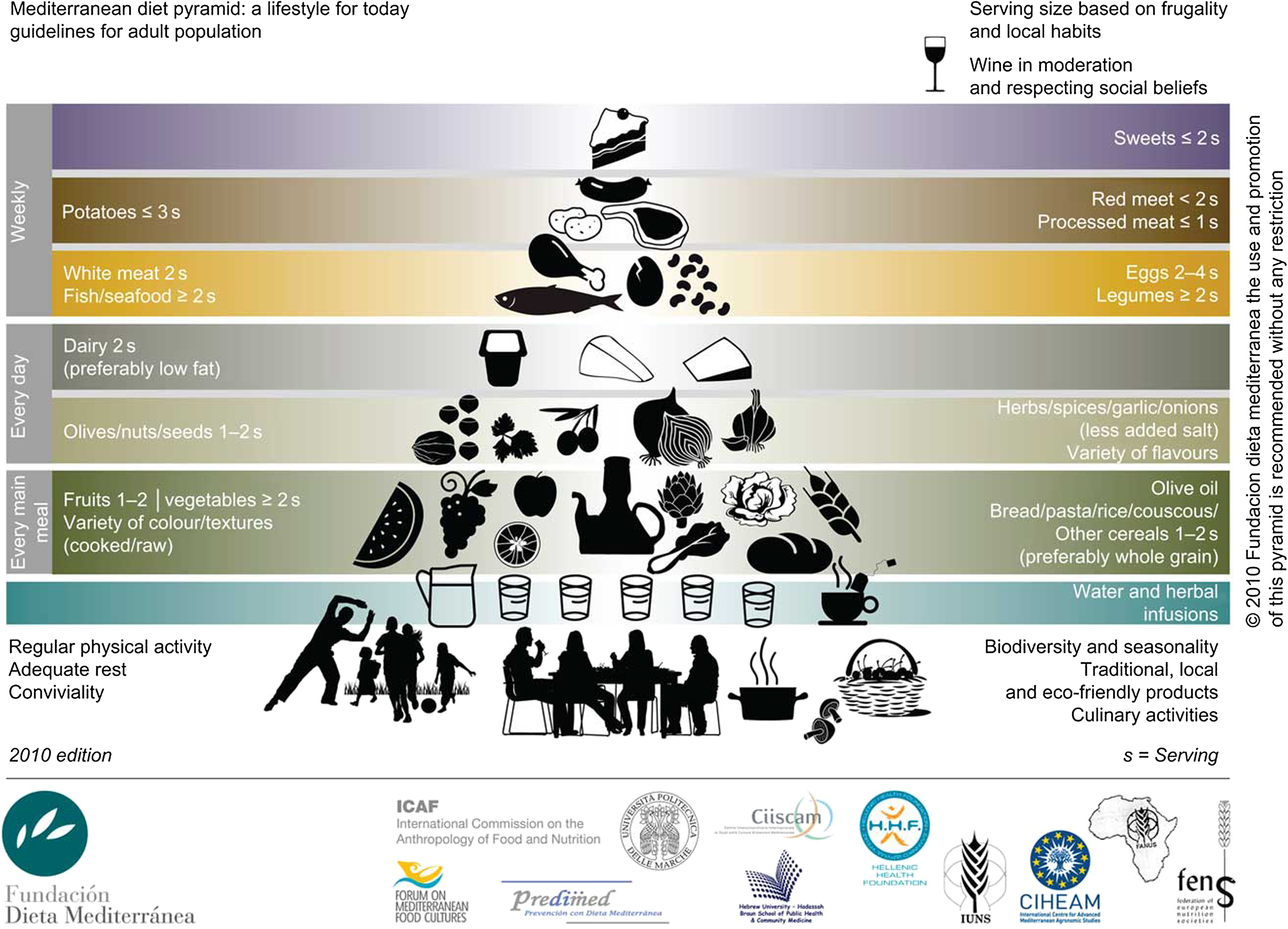

Supplement: Supplementary file 5 — Authors’ original file for figure 5 [file 12916_2014_994_MOESM5_ESM.tiff]

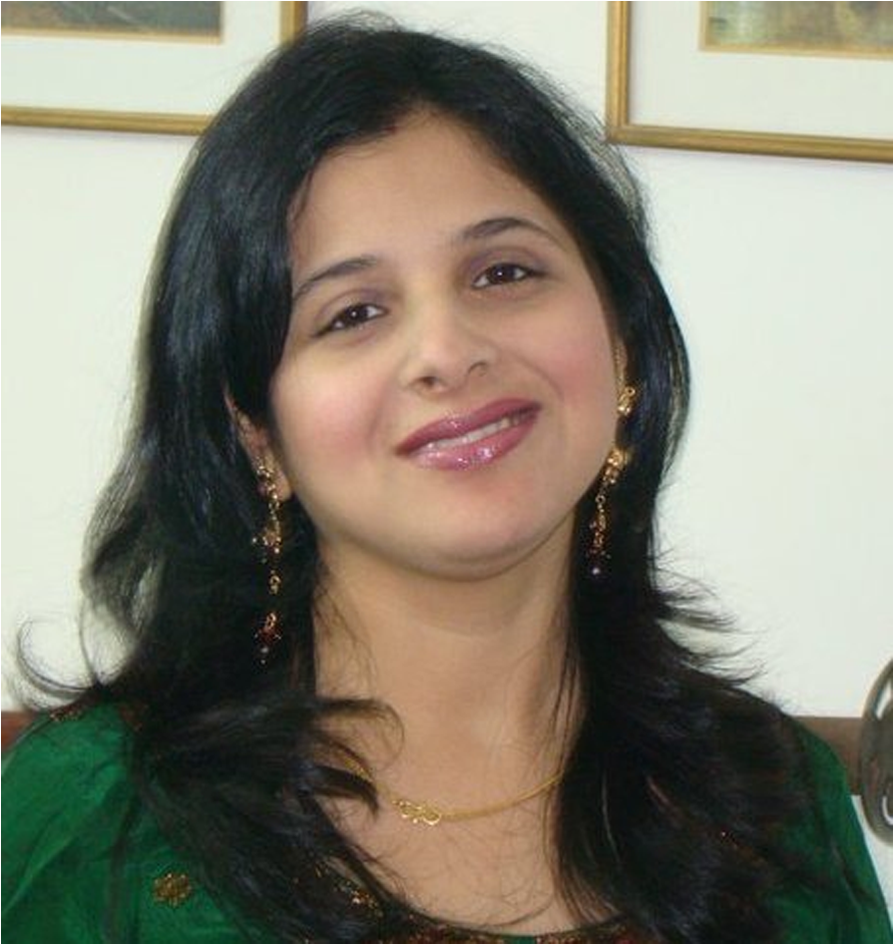

Supplement: Supplementary file 6 — Authors’ original file for figure 6 [file 12916_2014_994_MOESM6_ESM.tiff]

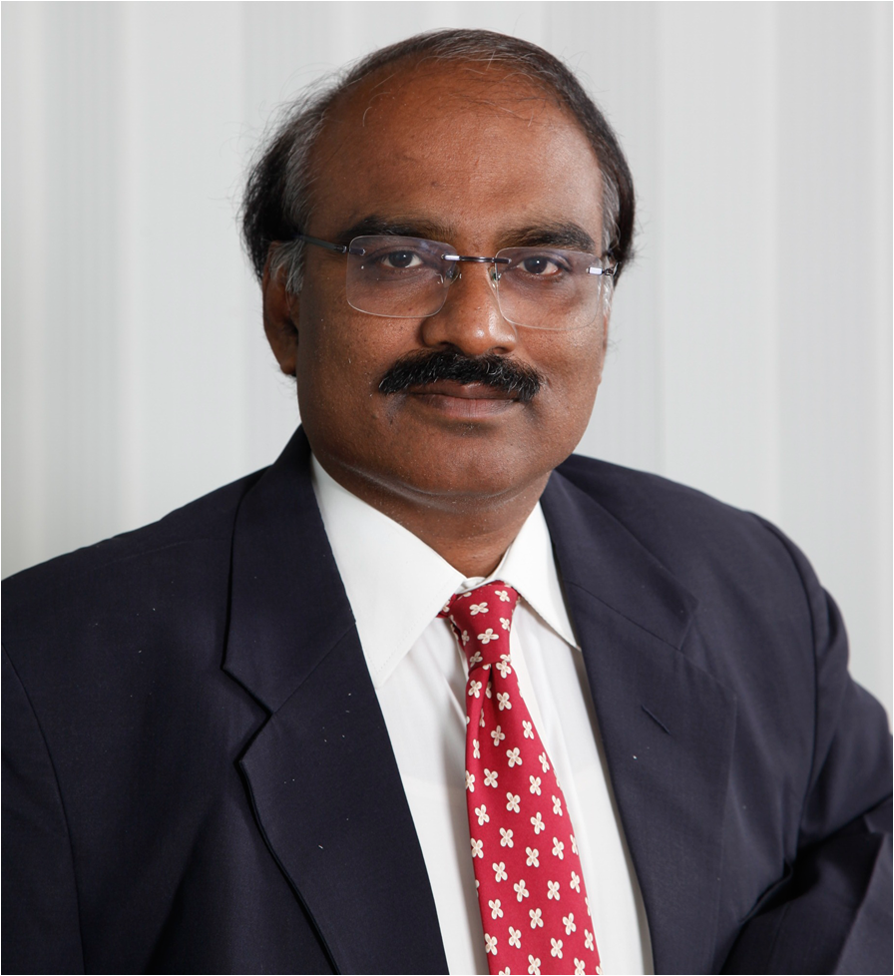

Supplement: Supplementary file 7 — Authors’ original file for figure 7 [file 12916_2014_994_MOESM7_ESM.tiff]

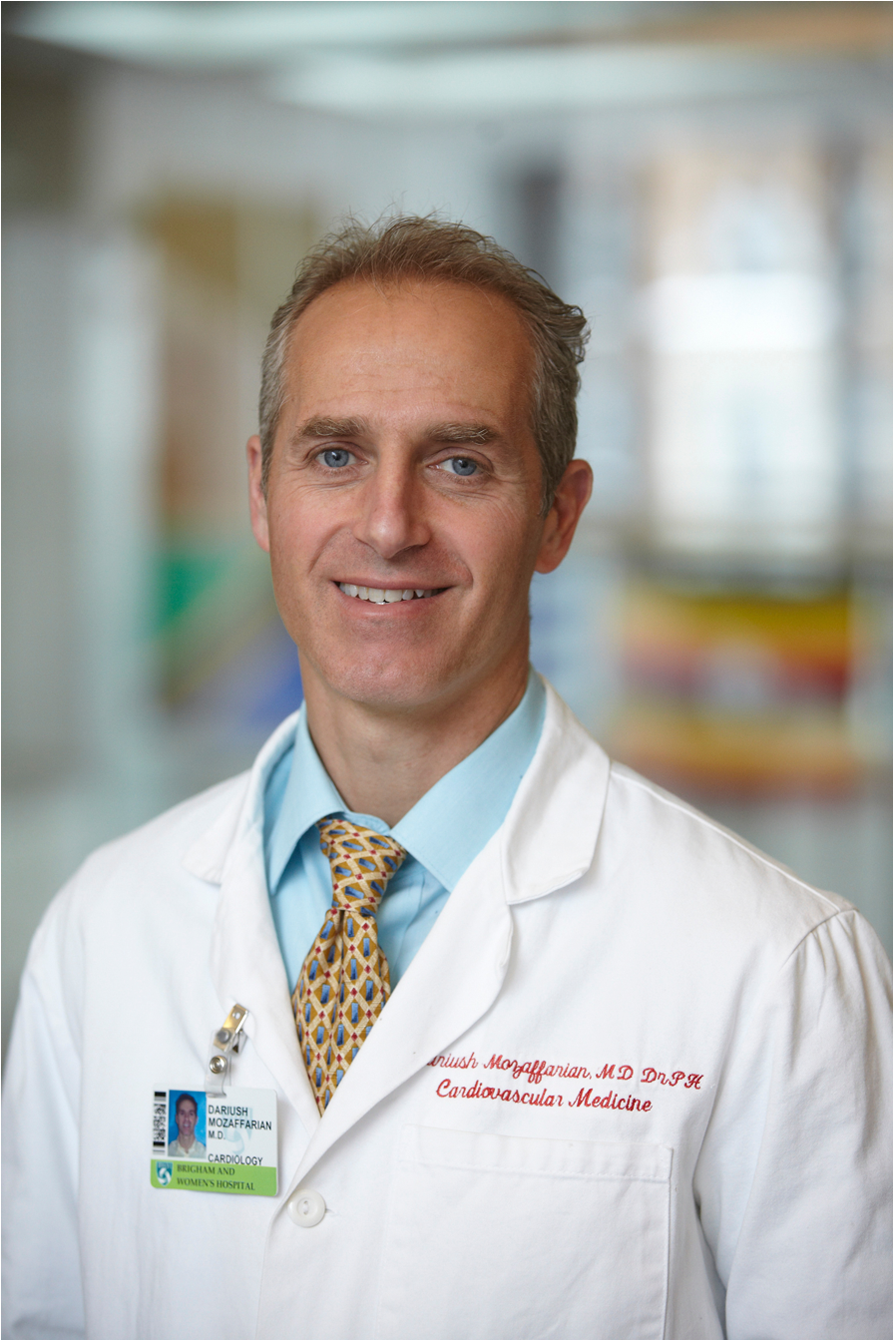

Supplement: Supplementary file 8 — Authors’ original file for figure 8 [file 12916_2014_994_MOESM8_ESM.tiff]

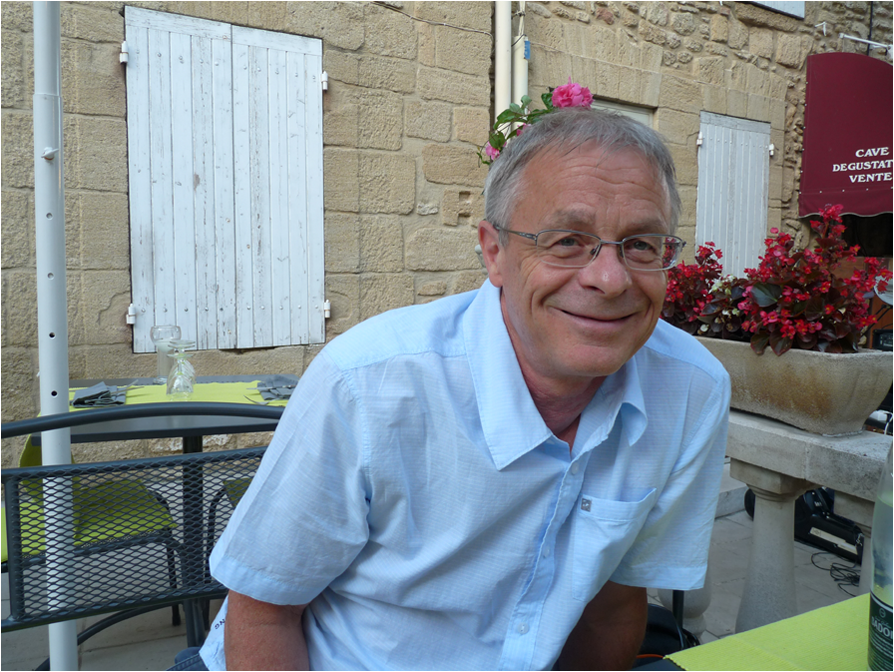

Supplement: Supplementary file 9 — Authors’ original file for figure 9 [file 12916_2014_994_MOESM9_ESM.tiff]
